# Enhancing Sustainability in Healthcare Delivery—A Challenge to the New Malaysia

**DOI:** 10.21315/mjms2019.26.1.1

**Published:** 2019-02-28

**Authors:** Dzulkefly Ahmad

**Affiliations:** Ministry of Health, Parcel E, Federal Government Administration Centre, 62590 Putrajaya, Malaysia

**Keywords:** health insurance, health indicator, health ministry, Malaysia, healthcare delivery

## Abstract

There have been substantial improvements in the health indicators since Malaysia achieved independence. These were accomplished through strong primary healthcare services addressing maternal and paediatric health, as well as the successful control of communicable diseases. The rate of decline in the mortality statistics has been at a virtual standstill, or at best, almost plateaued since 2000. However, with the plethora of national health issues at both the policy and delivery levels, we cannot continue on with ‘business as usual’. Therefore, we must strategise effective and practical approaches to a renewed and revamped national healthcare services for a modern ‘New Malaysia’ that are compatible with our quest toward the status of a ‘truly developed’ nation.

## Enhancing Sustainability in Healthcare Delivery—A Challenge to the New Malaysia

Since achieving independence, Malaysia has maintained a publicly-provided predominantly taxation-based healthcare system on the basis of need. It has been widely regarded as a successful healthcare system model, and it has been commended by the World Health Organization as a ‘low-cost health care system that provides universal and comprehensive services’ (1).

Arguably, some have suggested that Malaysia achieved universal healthcare coverage (UHC) as early as 1980s (2), albeit pertaining to mainly on life expectancy at birth, especially through the successful control of communicable diseases (CDs) and the significant improvements in maternal and child health (MCH). Thanks to the emphasis of the nation’s founding fathers’ emphasis on healthcare and educational spending, rather than ‘beefing up’ the armed forces.

The number of health facilities throughout the country has grown dramatically, from seven public clinics in 1957, to more than 1,000 (1167) in 1970 and again by more than 1,000 (2264) in 1980. This is equivalent to one new public clinic built every 3.8 days for the first 23 years of independence, when the population was at 14 million!

This network of public clinics, including both health and community clinics, together with public hospitals distributed relatively evenly within each district, and later, the tertiary or referral hospitals (state hospitals and specialist medical institutions) in bigger cities formed the backbone of the public health system (PHS).

Malaysia became a global benchmark of sorts for low and middle-income healthcare systems delivering equitable and effective health outcomes at a low cost with strong financial protection. This was achieved through public sector supply-side investments rather than demand-side financing schemes.

While the PHS achieved its purpose and mission of serving low income groups, with an emphasis on MCH and CD-related healthcare services, it deliberately left a gap or void in the health services sector for private facilities designed to serve the middle and upper-middle classes who were willing to pay for shorter wait times and better facilities. This dichotomous public-private healthcare construct soon evolved harmoniously, with private healthcare providers and insurers providing a contribution of approximately 48% of the total healthcare expenditures, which was equivalent to 2.2% of the gross domestic product (GDP).

Private healthcare facilities are regulated by the Ministry of Health (MOH) under the Private Healthcare Facilities and Services Act of 2006 (Act 586), and they are insured by Bank Negara Malaysia under the Financial Services Act of 2013 (3, 4). The government has encouraged further private investment by contracting out several services, namely drug distribution and hospital services, in addition to corporatising the National Heart Institute. Approximately 69% of the clinics (7,571 clinics) and 55% of the hospitals (200 hospitals with 14,799 beds) are privately owned. While the government itself currently has a total of 144 hospitals, including nine specialist medical institutions, like the National Cancer Institute and the Institute of Respiratory Medicine, it also owns several large private hospitals and hospital chains through Khazanah Nasional Berhad, its sovereign wealth fund.

Be that as it may, Malaysia’s earlier impressive successes are already showing signs of unsustainability with regard to changing lifestyle and demographic needs, fiscal sustainability and the financial risk protection afforded to the population, especially in underserved communities. More specifically, the findings of the Malaysia Health Systems Research Study (a collaboration between the Government of Malaysia and Harvard University) strongly suggest that Malaysia has a growing and dual burden of disease, with high and rapidly rising noncommunicable disease (NCD) rates alongside persistent problems with CDs, particularly dengue fever and tuberculosis (5).

International comparisons from peer comparators in the study suggest that greater improvements in the adult life expectancy could be achieved in Malaysia. Of particular concern is the fact that the vast majority of adults with diabetes mellitus (DM), hypertension and hypercholesterolemia are not aware of their conditions. Worryingly, this proportion has increased over the last nine years. Furthermore, of those who are aware of their DM diagnoses, only 38% of them had achieved a glucose level within the treatment range, creating doubt in the actual utilisation and effectiveness of patient care. This applies equally to other NCDs. There is little wonder that our health indicators have plateaued over the last decade, and we simply cannot continue doing the same things while hoping for better outcomes.

This brings us to a greater challenge. The question that begs an immediate response from the Government of the Day, and the Health Minister, in particular, is whether the healthcare model that has been successful in the past is viable or adept enough to handle the current and future health challenges of Malaysia? The challenge put to the Minister and his MOH is simply this: Towards achieving ‘Health for All’ (UHC), improving the health of the population and enhancing the quality of healthcare services in an affordable, responsive and sustainable manner, how can the current model of healthcare be tweaked or reformed?

Let us attempt to decipher these issues using a three-pronged approach: sustainable healthcare financing, the integration of healthcare services and the transformation of primary healthcare services. However, deliberations on the critical need for the integration of our healthcare services and the transformation of primary healthcare services will be dealt with on another occasion. Arguably, the toughest issue at hand is surely that of choosing a healthcare financing system that is worthy of the ‘New Malaysia’. Many of the analysts and top minds in the healthcare sector have alluded to the issue of the Malaysian healthcare system’s sustainability. A decade of underspending in the MOH’s budgetary allocation, which is now at 2.5% of the GDP (for the PHS), is well below the spending benchmark of an upper-middle income economy. A reasonable total healthcare expenditure would be 4.0% to 4.5% of the GDP. This has resulted in unending, inadequate and ageing public facilities epitomised by the matrix of the current bed to population ratio of 1.9:1,000 or approximately 1:525, which is well behind almost all of Malaysia’s regional peers. Additionally, the existing doctor to population ratio of 1:656 is also below the target of 1:400.

Despite the public healthcare sector inadequacies, rising treatment costs have made the public hospital admission rate twice that of the private sector. Subsequently, this has resulted in the perennial problems of congestion, heavy workloads and long waiting times in our public hospitals and clinics. Additionally, the greater workloads for public hospital specialists are largely due to the unequal distribution of specialists between private and public hospitals. Although 30% of the specialists are working in the public sector, they have treated 70% of the acute cases. Surely, public-private collaboration will be among the main priorities of the newly-minted Health Advisory Council that I have recently put into place, which is chaired by Tan Sri Dr Abu Bakar Sulaiman and his eminent team.

Moving forward, we must be serious about judiciously planning and addressing the health financing system. Currently, the financing sources include direct taxes, private insurance premiums, contributions to the Employees Provident Fund and the Social Security Organisation, indirect taxes and out-of-pocket payments, which equal 38.5% of the total health expenditure. After the failure of the Voluntary Health Insurance scheme proposed by the last government, many want the new government to follow the Social Health Insurance (SHI) model, or a hybrid or tweaked version of it. However, the advantages of SHI need to be examined against the Wagstaff Study for the World Bank, which opined that a tax revenue-funded system is more equitable and cost effective than SHI (6) This acrimonious subject has been debated for many years, but it now warrants further close examination by all relevant stakeholders before we can firmly arrive at the Malaysian model of a sustainable healthcare financing system.

Regardless, the new financing model should not only be designed to restructure the financial contribution sources, but it must also seek financial incentives for behavioural changes in the lifestyles and dietary habits of not just the high risk groups but the general population at large, to result in a drastic reduction in NCDs in the future, hopefully within our lifetime.
